# Incidental Finding of Subannular Perfused Aortic Root Abscess

**DOI:** 10.3390/diagnostics14010019

**Published:** 2023-12-21

**Authors:** Luise Vöhringer, Malte Niklas Bongers, Florian Helms, Aron Frederik Popov

**Affiliations:** 1 Department of Cardiothoracic and Vascular Surgery, Eberhard Karls University, 72074 Tuebingen, Germany; luise.voehringer@usb.ch; 2 Department of Cardiac Surgery, University Hospital Basel, 4031 Basel, Switzerland; 3 Department of Diagnostic and Interventional Radiology, Eberhard Karls University Tuebingen, 72074 Tuebingen, Germany; malte.bongers@med.uni-tuebingen.de; 4 Division for Cardiothoracic, Transplantation and Vascular Surgery, Hannover Medical School, 30625 Hannover, Germany; helms.florian@mh-hannover.de

**Keywords:** cardiac surgery, aortic surgery, computed tomography, endocarditis, aortic root abscess

## Abstract

An 83-year-old female presented with aortic valve stenosis requiring surgery, which was diagnosed with a transthoracic echocardiography three years ago. However, the patient declined the surgery at that time due to personal reasons. Three years later she presented again with signs of dizziness and weakness and progression of the aortic valve stenosis. Cardiac catheterization and a computed tomography scan were performed before the planned surgery. Surprisingly, a huge subannular perfused abscess hole around the aortic root companying a pericardial effusion was revealed. The patient underwent an urgent aortic root replacement with a tissue valve and an aortic ascending replacement without any complications. Intraoperative inspection confirmed an active aortic root and valve endocarditis.

##  

**Figure 1:**
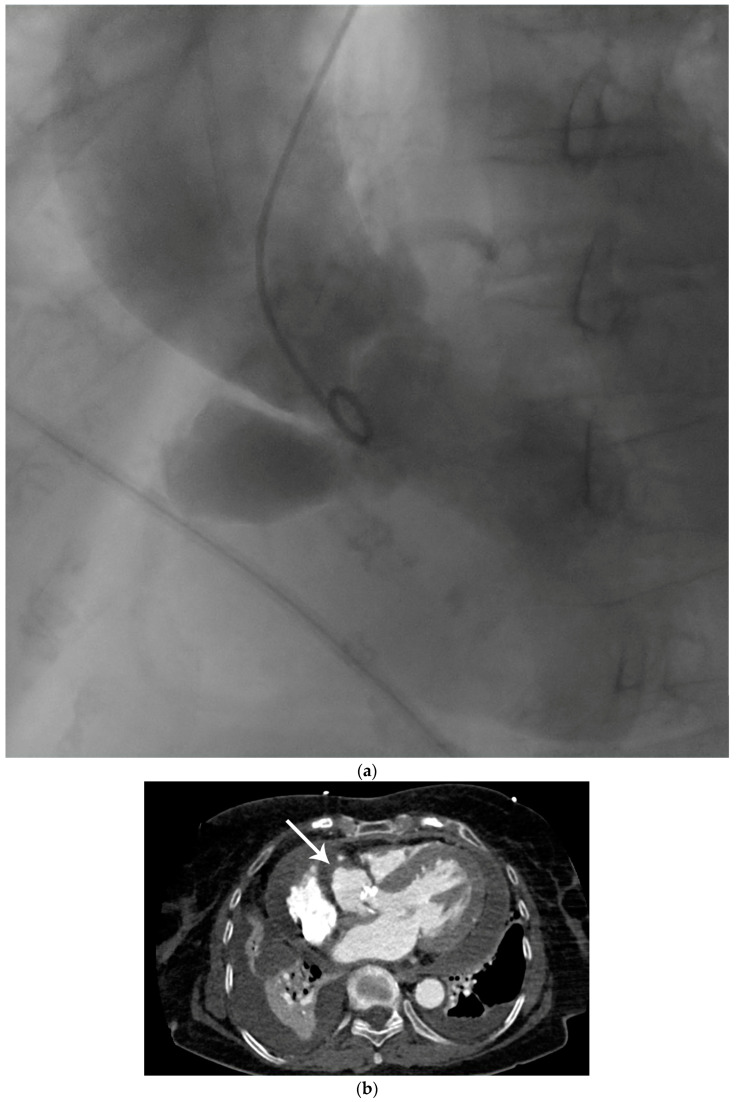

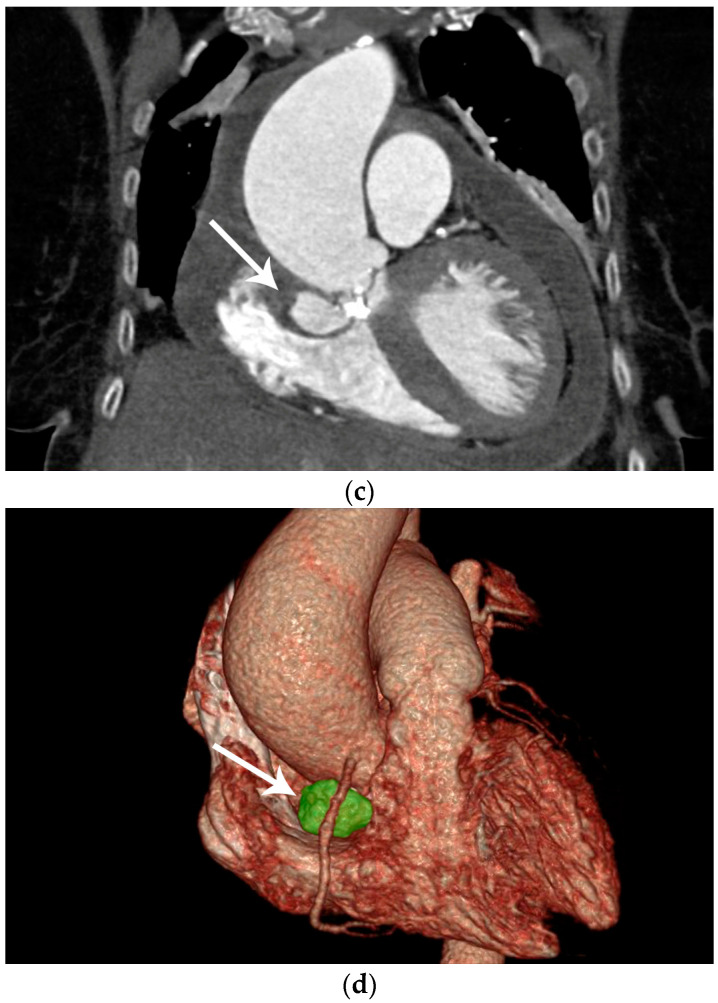
Preoperative diagnostic imaging of an 83-year-old female patient presenting with progressive symptomatic aortic valve stenosis. (**a**) Preoperative laevocardiography showed a para-aortic contrast media deposition. The cavity showed a wide base and was in continuity with the left ventricle. In this case, the initial diagnosis was made during standard laevocardiography. This allows for direct identification of the primary entry site and connection of the cavity, which is an important factor during differential diagnosis. If the initial diagnosis is made with other modalities such as echocardiography, the entity and connection of pericardiac and periaortic lesions may be more difficult. As an example, Emmert et al. reported a case of a chronic subannular abscess that was misdiagnosed for a sinus of Valsalva aneurysm and was only diagnosed correctly during intraoperative exploration [1]. Thus, live transcatheter contrast imaging as performed here can help to avoid incorrect preoperative diagnosis and preparation. In this case, preoperative echocardiography remained inconclusive with respect to the suspected lesion, although transthoracic or transesophageal echocardiography is widely accepted as the primary imaging method for infectious cardiac diseases [2,3]. In this selected patient group, in which the topographic anatomy remains unclear in other imaging modalities, laevocardiography may provide critical additional information for planning of the procedure. However, the risk of embolization associated with catheterization must be taken into account, particularly in the case of larger vegetations. (**b**) To confirm the initial preliminary diagnosis, contrast-enhanced computed tomography was utilized. Computed tomography, or cardiac magnetic resonance imaging, has increasingly become the gold standard for the imaging of more complex complications of infective cardiac diseases [4]. The main advantage of this imaging technique is the three-dimensional visualization of the cavity dimensions and connections. For this, electrocardiographic gating during computed tomography is recommended to avoid artifacts. In this horizontal plane of the computed tomography imaging of our patient, the wall of the spurious aneurysm was thickened (arrow), which may correspond to partial mural thrombosis or post-infective morphologic changes. In this context is should be considered that aortic subannular abscess-like formations can arise not only from infective endocarditis, but may also result from non-infective inflammatory lesions such as Libman–Sacks endocarditis, as reviewed by Murillo et al. [4]. Additionally, left ventricular hypertrophy, pericardial effusion, and pleural effusion were apparent in computed tomography imaging, indicating the beginning of cardiac decompensation. (**c**) In the frontal plane, the connection of the abscess cavity to the left ventricular outflow tract was visualized. Heavy calcification not only of the aortic valve but also of the base of the pseudoaneurysm was noted. Additionally, the aneurysmatic dilatation of the ascending aorta, which most likely resulted from the chronic aortic stenosis as a poststenotic aneurysm, can be seen in this plane. In prior studies, this poststenotic dilatation has been described for aortic stenosis, especially when the aortic valve is bicuspid [5]. (**d**) Three-dimensional reconstruction of the suspected lesions may be of great interest for operative planning, since this technique allows for the visualization of the local topography of the target lesion. In this case, the close topographic proximity of the abscess excavation to the right coronary artery became apparent (arrow). With ever improving rendering and electrocardiographic gating techniques, contrast-enhanced computed tomography has evolved into a powerful diagnostic tool in the preoperative planning for complex cardiac malformations [6].

## Data Availability

Data is unavailable due to privacy and ethical restrictions.
